# Active and sedentary behaviors in youth (6–14 years old): Data from the IAN-AF survey (2015–2016)

**DOI:** 10.1097/j.pbj.0000000000000161

**Published:** 2022-02-08

**Authors:** Andreia Nogueira Pizarro, Daniela Correia, Carla Lopes, Pedro J. Teixeira, Jorge Mota

**Affiliations:** a Research Center in Physical Activity and Health (CIAFEL), Faculty of Sport, University of Porto, Porto, Portugal; b Public Health and Forensic Sciences, and Medical Education Department, Faculty of Medicine, University of Porto, Porto, Portugal; c EPIUnit, Institute of Public Health, University of Porto, Porto, Portugal; d Interdisciplinary Study Centre for Human Performance (CIPER), Faculty of Human Kinetics (FMH), University of Lisbon, Cruz Quebrada, Portugal.

**Keywords:** national prevalence, outdoor play, physical activity, TV time

## Abstract

**Background:**

Strategic health interventions must be guided by effective surveillance systems that monitor population trends and patterns, therefore we aimed to provide youth's (6–14 years) national prevalence estimates of physical activity (PA), active outdoor play (AOP), sedentary behaviur (SB), and structured PA (SPA).

**Methods:**

Measures of moderate-to-vigorous PA, SB, AOP, and SPA, were obtained for 719 participants in 2 groups (6–9 and 10–14 years) using diaries and questionnaires. National estimates were calculated, by sex, region, and parental education.

**Results:**

While 54% (95%CI: 47.4–61.3) of youth meet PA recommendations, the prevalence is lower for the older group (*P* *=* .034). Similarly, AOP decreases with age both on weekends, from 94.3% (95%CI: 90.8–97.8) to 68.7% (95%CI: 62.2–75.1), and on weekdays from 84% (95%CI: 76.5–91.5) to 64.8% (95%CI: 59–70.6). Watching TV for ≥2 h/day is extremely high on weekends [71.3% (95%CI: 65.4–77.2)] and more prevalent in boys than girls on weekdays (*P* = .043). Higher parental education significantly increased SPA and active time. Time in SB was greater in boys [7.26 h (95%CI: 6.87–7.65)] than girls [6.48 h (95%CI: 6.09–6.87)] and increased with age (*P* < .001).

**Conclusions:**

Almost half of the youth failed to achieve PA guidelines, whereas 71% exceeded recommended TV time on weekends, suggesting the urgency of intervention measures.

## Introduction

The decreased opportunities for physical activity (PA) and the progressive time spent on screen-based and sedentary leisure activities increased concerns about lifestyles promoting non-communicable diseases, particularly obesity, among youth.^1^

Regular PA, of at least moderate intensity, is associated with extensive health benefits^2^ and active outdoor play (AOP) is a simple way suggested to increase children's PA.^3^ When children play outside they are more active and spend less time sedentary compared to when they are indoors.^3^ Furthermore, research reveals adverse health outcomes of sedentary behaviors (SB) during childhood and suggests that >2 h/day of TV viewing relates to less physical and psychosocial health.^4^ Moreover, physiological benefits from reducing SB seems different from those of increasing moderate-to-vigorous PA (MVPA).^5^ Worldwide recognition of such evidence is expressed in the PA recommendations widespread by international public health authorities^6,7^ suggests that children and adolescents (5–17 years) should achieve at least 60 minutes of MVPA/daily^7^ and set limits to screen time.^6^ Unfortunately, most children are far from accomplishing these recommendations.^8,9^ Moreover, few global repositories of descriptive PA for elementary school children exist, and there is still much to learn on the PA levels of younger age groups.

Prior estimates for PA on Portuguese population were conducted several years ago,^10^ only for children above 9 years old^9,11^ and report alarmingly low percentages for achieving MVPA recommendations. Instead, watching television ≥2 hours on weekdays ranges from 45% to 62%. Besides, a recent report highlight inconsistencies in PA data and a lack of robust information, addressing the need to build an effective national surveillance system^12^ to monitor trends and patterns of change over time, guiding health interventions.

Therefore, our aim is to describe the national prevalence estimates of active and SB in Portuguese youth between 6 and 14 years old using data from National Food, Nutrition and Physical Activity Survey (IAN-AF).^13^

## Methods

In the scope of the IAN-AF survey, a representative sample of the Portuguese population from 3 to 84 years old was selected by multistage sampling, stratified by geographical region (NUTS II), using as a sampling frame the national health registry. A total of 6553 individuals (participation rate among eligible: 33.4%, 52.6% in children) accepted the first face-to-face interview using computer-assisted personal interviewing (CAPI) from October 2015 to September 2016. Detailed information is described elsewhere.^13^

For our analysis, 719 participants (368 girls, 50.3%) aged 6 to 14 years with information on AOP and SB were included, and 592 (304 girls, 50.3%) completed PA diaries. The e-module “Move” from an electronic platform (You eAT & Move) assisted PA data collection and management.

### Socioeconomic data

Datasets from the National Health registries allowed to collect age and gender. The difference between the assessment date and birth determined participants’ age.

Educational level defined as the highest number of completed school years of one of the parents was collapsed into 3 categories: less than secondary; secondary and post-secondary; and tertiary education.

Participants were allocated to the statistical Geographical Units **—** NUTS II (North, Centre, Lisbon Metropolitan Area, Alentejo, Algarve, Madeira, Azores) of their Primary Health Care Unit.

### PA and sedentary time

A self-report activity diary with a grid dividing each day (24 h) into 15 minutes’ periods, adapted from Bouchard 3-days Activity diary,^14^ was used to assess PA and SB.

Parents (6–9 years) or children (10–14 years) reported the primary activity performed in each – 15-minute interval over 2 consecutive weekdays and 2 weekend days. Each activity was converted into metabolic ratios of expended energy as suggested in the PA compendium.^15^ Energy expenditure was estimated by multiplying the related metabolic equivalent of task (MET) by the time spent in each activity (min/day) and individual daily expenditure was computed as the mean expenditure of the 4-day diaries. MVPA was defined as ≥3 METs.^16^

### TV time, AOP, and structured PA

TV time, AOP, and structured PA (SPA) were assessed by questionnaire. Parents (6–9 years) or children (10–14 years) were asked: in an average week/weekend day how much time “does your children/do you” spend watching TV; playing actively (eg, playing in the park, playing soccer, riding a bike, running, and walking outside).” The possible answers were: none, <15 minutes; 30 minutes, 1 hour, 2 hours, 3 hours, 4 hours, 5 hours, and ≥6 hours. SPA was accessed by a yes or no question: “Do you take part in regular physical activity or sports?.”

### Ethics

Ethical approval was obtained from the National Commission for Data Protection, the Ethical Committee of the Institute of Public Health of University of Porto, and from the Ethical Commissions of each Regional Administrations of Health.

Written informed consent was obtained for all participants according to the Ethical Principles for Medical Research involving human subjects expressed in the Declaration of Helsinki^17^ and the national legislation. As our sample was composed of children and adolescents, written agreements from the parents were required. The participants with 10 or more years old were asked to sign the consent form together with their legal representative. Documents with identification data were treated separately and stored in a different dataset.

### Statistics

Multistage complex sample design weighted estimates according to Portuguese population distribution by age, sex, and geographical region. Variables of interest were evaluated by sex, age group, parents’ education, and geographical regions. Differences in mean MVPA time/day and mean sedentary time/ day between groups were tested using one-way analysis of variance. The χ^2^-test evaluated differences between groups in the prevalence of achieving ≥60 min/day of MVPA, ≥60 min/day of AOP, participation in SPA, and prevalence of watching TV ≥2 h/ day.

Logistic regression was conducted to analyze associations between children's characteristics and healthy behaviors with achieving ≥60 min/day in MVPA and with participation in SPA.

The association between engagement in ≥60 min/day in MVPA and participation in SPA with socioeconomics and healthy behaviors were evaluated using weighted logistic regressions, obtaining crude and adjusted odds ratio (OR). Gender, age group, and parents’ education (model 2), as well as sleep hours and quantiles of energy intake (model 3) and sleep hours and Body mass index (BMI) categories (model 3) were included as confounders. Given that only 1.2% of the participants were underweight, we combined underweight and normal weight in 1 category for statistical reasons.

A significance level of 5% and independence between observations were assumed. Analyses were performed in 2018 in R software (The R Project for Statistical Computing), using the library “survey.”

## Results

The information of 719 participants was recorded using the questionnaire, and 592 participants using diaries. Approximately half of the children were of each gender and around 60% were between 10 and 14 years old. The most prevalent parental education was secondary and post-secondary education (47.1%) and only 15% had less than secondary education. The distribution of the sub-sample characteristics was similar to the total sample (Table 1).

**Table 1:** Characteristics of the children based on the total sample and in the sub-sample with physical activity diaries.

	Total sample from 6 to 14 years old			Sub-sample with physical activity diaries		
		
		Estimated population			Estimated population	
				
	Sample size	n	%	Sample size	n	%
*Total*	719	826,712	100.0%	592	826,869	100.0%
*Gender*						
Girls	368	416,167	50.3%	304	416,167	50.3%
Boys	351	410,545	49.7%	288	410,702	49.7%
*Ages*						
6–9 years old	302	319,193	38.6%	245	319,350	38.6%
10–14 years old	417	507,519	61.4%	347	507,519	61.4%
*Region*						
North	110	274,689	33.2%	71	274,689	33.2%
Centre	137	164,338	19.9%	126	164,338	19.9%
Lisbon	101	238,890	28.9%	94	238,890	28.9%
Alentejo	80	56,958	6.9%	76	56,958	6.9%
Algarve	83	41,940	5.1%	76	41,940	5.1%
Madeira	102	24,225	2.9%	86	24,382	2.9%
Azores	106	25,672	3.1%	63	25,672	3.1%
*Parents education*						
Less than secondary education	108	124,042	15.0%	73	124,042	15.0%
Secondary and post-secondary education	356	389,710	47.1%	298	389,711	47.1%
Tertiary education	255	312,960	37.9%	221	313,116	37.9%

The prevalence of Portuguese children accomplishing ≥60 min/ day of MVPA (Table 2) at a national level is 54.4% (95%CI: 47.4–61.3). Significant differences were found between age groups with 61.5% (95%CI: 52.5–70.6) of 5–9 years old vs 50.1% (95%CI: 41.7–58.4) of the 10–14 years old meeting PA recommendations.

**Table 2:** Active and sedentary habits at national level stratified by gender, age group, and parents’ education.

		Total	Gender			Age			Parents’ education			
								
			Girls	Boys	*P* value	6–9 years	10–14 years	*P* value	Less than secondary	Secondary and post-secondary	Tertiary	*P* value
*Physical activity*												
*MVPA time (≥ 3MET/h, in hours)*												
	mean	1.40	1.29	1.52	.088	1.53	1.31	.084	**1.09**	**1.44**	**1.38**	**.027**
	95%CI	(1.22–1.57)	(1.08–1.50)	(1.29–1.74)		(1.33–1.73)	(1.10–1.53)		**(0.77–1.41)**	**(1.19–1.68)**	**(1.15–1.62)**	
≥ *60 min/MVPA (*≥ *3MET/h)*												
	%	54.4	50.7	58.4	.161	**61.5**	**50.1**	**.034** ^**∗**^	39.7	54.9	55.1	.278
	95%CI	(47.4–61.3)	(41.1–60.3)	(50.6–66.2)		**(52.5–70.6)**	**(41.7–58.4)**		(24.5–54.9)	(46.4–63.4)	(44.2–66.1)	
*≥ 60 min Active outdoor play*												
Weekdays	%	72.2	70.3	74.2	.433	**84.0**	**64.8**	**.001** ^**∗∗**^	74.6	71.1	72.7	.878
	95%CI	(67.7–76.7)	(64.0–76.5)	(67.2–81.3)		**(76.5–91.5)**	**(59.0–70.6)**		(62.9–86.3)	(64.9–77.3)	(64.6–80.7)	
Weekends	%	78.6	74.9	82.3	.062	**94.3**	**68.7**	**<.001** ^**∗∗∗**^	72.8	77.8	81.9	.395
	95%CI	(74.5–82.6)	(69.4–80.3)	(76.7–88.0)		**(90.8–97.8)**	**(62.2–75.1)**		(61.7–83.8)	(71.5–84.0)	(74.7–89.0)	
*Structured physical activity*												
	%	60.3	57.6	63.1	.244	62.0	59.3	.618	**39.0**	**51.8**	**79.5**	**<.001** ^**∗∗∗**^
	95%CI	(55.3–65.4)	(50.4–64.9)	(56.6–69.6)		(53.5–70.5)	(53.1–65.5)		(26.8–51.3)	**(45.3–58.3)**	**(73.0–85.9)**	
*Sedentary behavior*												
*Sedentary time (<1.6 MET/h, in hours)* ^a^												
	mean	**6.85**	**6.48**	**7.26**	**.001** ^**∗∗**^	**5.90**	**7.42**	**<.001** ^**∗∗∗**^	7.64	6.77	6.78	.055
	95%CI	**(6.54–7.16)**	**(6.09–6.87)**	**(6.87–7.65)**		**(5.51–6.29)**	**(7.02–7.82)**		(6.90–8.39)	(6.42–7.11)	(6.24–7.31)	
≥ *120 min TV time*												
Weekdays	%	**34.8**	**30.0**	**39.6**	**.043** ^**∗**^	34.5	35.0	.904	42.4	37.9	27.9	.065
	95%CI	**(28.9–40.7)**	**(22.8–37.3)**	**(32.2–47.1)**		(27.3–41.7)	(27.8–42.2)		(28.0–56.8)	(30.0–45.9)	(21.0–34.8)	
Weekends	%	71.3	67.5	75.1	.167	74.7	69.1	.255	75.7	73.4	66.9	.392
	95%CI	(65.4–77.2)	(59.4–75.7)	(67.6–82.5)		(68.0–81.4)	(61.3–77.0)		(62.3–89.1)	(66.0–80.7)	(58.3–75.6)	

Boldface indicates statistical significance (^∗^*P* < .05, ^∗∗^*P* < .01, ^∗∗∗^*P* < .001). CI, confidence interval, 95%; MVPA: moderate to vigorous physical activity; TV, television.

a Sedentary time (excluding sleeping time and class time).

Average MVPA time was 84 minutes, lower in children with less-educated parents (1.09 h; 95%CI: 0.77–1.41). Children from more educated parents presented significantly higher participation on SPA (79% vs 39%).

Table 3 shows the distribution of meeting MVPA guidelines according to geographical regions NUTs II. No significant differences were found for achieving PA guidelines. However, significant differences in MVPA time were found for NUTs II. The North region reported about 1 h/day (1.03 h; 95%CI: 0.78–1.28), while Azores had the highest MVPA time (2.16 h; 95%CI: 1.53–2.78).

**Table 3:** Active and sedentary habits of the Portuguese children, stratified by region (NUT II).

		North	Centre	Lisbon MA	Alentejo	Algarve	Madeira	Azores	*P* value
*Physical activity*									
*MVPA time (≥ 3MET/h, in hours)*									
	Mean	1.03	1.36	1.44	1.82	1.91	1.76	2.16	**.021** ^**∗**^
	95%CI	(0.78–1.28)	(0.96–1.77)	(1.07–1.82)	(1.45–2.19)	(1.39–2.44)	(1.17–2.35)	(1.53–2.78)	
*≥ 60 min/MVPA* ^**∗**^ *(≥ 3MET/h)*									
	%	43.7	51.4	57.5	67.3	66.1	67.6	75.9	.115
	95%CI	(28.4–58.9)	(38.6–64.1)	(43.0–72.0)	(59.2–75.4)	(50.4–81.7)	(51.3–84.0)	(66.4–85.5)	
≥ *60 min Active outdoor play*									
Weekdays	%	58.5	70.9	87.8	85.7	64.3	68.1	69.0	**<.001** ^**∗∗∗**^
	95%CI	(48.1–69.0)	(63.9–77.8)	(82.0–93.7)	(78.2–93.2)	(46.9–81.7)	(59.8–76.3)	(52.9–85.2)	
Weekends	%	78.6	71.3	81.1	89.9	82.0	68.5	80.5	.157
	95%CI	(70.2–87.1)	(63.4–79.1)	(73.4–88.8)	(82.4–97.4)	(73.9–90.1)	(56.2–80.7)	(66.0–95.1)	
*Structured physical activity*									
	%	63.4	57.6	56.3	61.6	73.5	61.8	56.8	.412
	95%CI	(55.6–71.3)	(48.8–66.5)	(43.0–69.6)	(50.8–72.5)	(64.0–83.0)	(55.6–67.9)	(53.5–60.1)	
*Sedentary behavior*									
*Sedentary time (<1.6 MET/h, in hours)* ^a^									
	mean	6.32	6.47	7.64	6.93	6.15	7.03	7.26	**.007** ^**∗∗**^
	95%CI	(5.74–6.90)	(6.02–6.92)	(6.88–8.39)	(6.45–7.40)	(5.77–6.54)	(6.33–7.73)	(6.52–8.00)	
*≥ 120 min TV time*									
Weekdays	%	27.7	33.9	39.3	42.0	36.4	45.3	45.7	.304
	95%CI	(17.8–37.6)	(23.6–44.3)	(24.9–53.8)	(30.3–53.8)	(20.5–52.2)	(37.3–53.3)	(40.2–51.1)	
Weekends	%	69.3	77.3	67.6	76.5	79.0	68.6	67.4	.491
	95%CI	(55.7–82.8)	(72.2–82.4)	(55.5–79.7)	(65.3–87.6)	(70.1–87.8)	(63.4–73.8)	(55.9–79.0)	

Boldface indicates statistical significance (^∗^*P* < .05, ^∗∗^*P* < .01, ^∗∗∗^*P* < .001). CI, confidence interval, 95%; MA, metropolitan area; MVPA, moderate-to-vigorous physical activity; TV, television.

a Sedentary time (excluding sleeping time and class time).

AOP for ≥60 min/day was reported by 72.2% ofchildren. AOP decreases significantly with age from 84% (95%CI: 76.5–91.5) at 6–9 years old to 64.8% (95%CI: 59–70.6) at 10–14 years on weekdays and even markedly on weekends [94.3% (95%CI: 90.8–97.8) vs 68.7% (95%CI: 62.2–75.1)]. Differences across regions were found on AOP on weekdays as Lisbon Metropolitan area had a prevalence of 87.8% (95%CI: 82.0–93.7), significantly higher than North, Center, Algarve, or Madeira.

The prevalence of SPA was 60.34% (95%CI: 55.3–65.4) and no differences for gender, age groups, or across regions were found. However, 79.4% (95%CI: 73–85.9) of children from parents with tertiary education, significantly higher than 51.78% (95%CI: 45.3–58.3) from parents with secondary and 39.04% (95%CI: 26.8–51.3) less than secondary education were involved in SPA.

Portuguese children spent ≈7 h/day in SB (excluding sleep and school time). Sedentary time (<1.6 MET/h), was higher in boys (7 h 15 min; 95%CI: 6.87–7.65) compared to girls (6 h 30 min; 95%CI: 6.09–6.87) and increased with age from around 6 h/day in the younger group to 7 h 25 min/day in the older group. Significant differences between regions (*P*-value = .007) were also found. Participants from Lisbon metropolitan area reported more SB (7 h 38 min) while participants from Algarve spent the lowest time in SB (6 h 10 min).

The prevalence of ≥2 h/day watching TV on weekends is 71.3% (95%CI: 65.4–77.2) while on weekdays is 34.8% (95% CI: 28.9–40.7). About 40% (95%CI: 32.2–47.1) of boys spent ≥2 h/day watching TV on weekdays, marginally different from 30% (95%CI: 22.8–37.3) reported in girls.

Association between children's characteristics and healthy behaviors with achieving ≥60 min/day in MVPA and with SPA are presented in Tables 4 and 5, respectively. The crude models show age as the only significant predictor for achieving MVPA recommendations. After adjusting for gender, parent's education, sleep hours, and BMI, older participants had 50% lower odds of achieving MVPA recommendations than their younger peers (OR, 0.50; 95%CI: 0.32–0.80).

**Table 4:** Associations between achieving MVPA recommendations, with socioeconomic and health-related indicators of Portuguese children

	≥ *60 min/MVPA n (%)*	Model 1^∗^ OR (95%CI)	Model 2^†^ OR (95%CI)	Model 3^‡^ OR (95%CI)	Model 4^§^ OR (95%CI)
*Gender*					
Girls	171 (50.7)	Ref	Ref	Ref	Ref
Boys	190 (58.4)	1.30 (0.85, 1.99)	1.41 (0.94, 2.13)	1.32 (0.88, 1.98)	1.42 (0.94; 2.15)
*Age group*					
6–9 years old	168 (61.5)	Ref	Ref	Ref	Ref
10–14 years old	193 (50.1)	**0.55 (0.35, 0.84)**	**0.52 (0.33, 0.82)**	**0.50 (0.31, 0.78)**	**0.50 (0.32; 0.80)**
*Parents education*					
Less than secondary education	37 (39.7)	Ref	Ref	Ref	Ref
Secondary and post-secondary education	181 (54.9)	1.98 (1.00, 3.92)	1.84 (0.93, 3.61)	1.82 (0.95, 3.49)	1.85 (0.93; 3.70)
Tertiary education	143 (55.1)	2.02 (0.92, 4.43)	1.77 (0.82, 3.82)	1.77 (0.82, 3.78)	1.81 (0.82; 3.99)
*Living with both parents*					
No	56 (53.6)	Ref	Ref	Ref	Ref
Yes	305 (54.5)	0.93 (0.50, 1.74)	0.83 (0.45, 1.54)	0.84 (0.46, 1.51)	0.83 (0.45; 1.53)
*Region type*					
Mostly urban	263 (52.8)	Ref	Ref	Ref	Ref
Moderately urban	54 (58.6)	1.41 (0.63, 3.15)	1.28 (0.60, 2.74)	1.30 (0.60, 2.83)	1.36 (0.64; 2.90)
Mostly rural	43 (59.3)	1.29 (0.60, 2.76)	1.37 (0.61, 3.10)	1.45 (0.62, 3.37)	1.45 (0.65; 3.21)
*BMI category*					
Underweight/normal weight	235 (55.7)	Ref	Ref	Ref	Ref
Pre-obesity	82 (50.8)	0.79 (0.50, 1.24)	0.77 (0.46, 1.27)	0.78 (0.47, 1.30)	0.77 (0.46; 1.27)
Obesity	44 (57.0)	1.00 (0.45, 2.22)	1.01 (0.46, 2.23)	1.02 (0.46, 2.25)	1.01 (0.46; 2.24)
*Sleep hours*					
<9.5 h/night	60 (51.3)	Ref	Ref	Ref	Ref
9.5–11 h/night	215 (55.0)	1.16 (0.69; 1.95)	1.05 (0.61; 1.83)	1.13 (0.65, 1.96)	1.07 (0.61; 1.88)
≥11 h/night	86 (55.5)	1.19 (0.58; 2.44)	0.99 (0.49; 2.02)	1.05 (0.52, 2.11)	0.99 (0.49; 2.02)
*5/day fruits and vegetables*					
No	275 (52.0)	Ref	Ref	Ref	Ref
Yes	86 (61.4)	1.49 (0.81, 2.73)	1.37 (0.73, 2.55)	1.26 (0.69, 2.29)	1.26 (0.67; 2.35)
*Energy (quartiles of intake)*					
1 (lower intake)	82 (48.1)	Ref	Ref	Ref	Ref
2	87 (50.5)	1.17 (0.55, 2.48)	1.13 (0.52, 2.49)	1.12 (0.50, 2.50)	1.15 (0.52, 2.57)
3	96 (62.2)	1.51 (0.73, 3.11)	1.56 (0.73, 3.32)	1.58 (0.75, 3.31)	1.66 (0.78, 3.53)
4 (higher intake)	96 (54.9)	1.28 (0.59, 2.77)	1.49 (0.68, 3.26)	1.50 (0.69, 3.29)	1.55 (0.70, 3.42)

Boldface indicates statistical significance. BMI, body mass index; MVPA, moderate-to-vigorous physical activity.

∗ Crude model.

† Model adjusted to gender, age, and parents’ education.

‡ Model adjusted to gender, age, parents’ education, sleep hours, and energy intake.

§ Model adjusted to gender, age, parents’ education, sleep hours, and BMI categories.

**Table 5:** Associations between participation in SPA with socioeconomic and health-related indicators of Portuguese children.

	Structured PA n (%)	Model 1^∗^ OR (95%CI)	Model 2^†^ OR (95%CI)	Model 3^‡^ OR (95%CI)	Model 4^§^ OR (95%CI)
*Gender*					
Girls	215 (56.6)	Ref	Ref	Ref	Ref
Boys	226 (63.1)	1.26 (0.86; 1.85)	1.39 (0.91; 2.14)	1.65 (0.98; 2.79)	**1.71 (1.02; 2.88)**
*Age group*					
6–9 years old	188 (62.0)	Ref	Ref	Ref	Ref
10–14 years old	253 (59.3)	0.89 (0.57; 1.39)	1.01 (0.63; 1.63)	1.09 (0.58, 2.06)	1.10 (0.60; 2.03)
*Parents education*					
Less than secondary education	43 (39.0)	Ref	Ref	Ref	Ref
Secondary and post-secondary education	198 (51.8)	1.68 (0.97; 2.91)	1.69 (0.98; 2.92)	**2.33 (1.21, 4.48)**	**2.41 (1.24; 4.69)**
Tertiary education	200 (79.5)	**6.04 (3.15; 11.58)**	**6.25 (3.33; 11.73)**	**8.68 (4.10, 18.38)**	**8.28 (3.95; 17.37)**
*Living with both parents*					
No	68 (52.7)	Ref	Ref	Ref	Ref
Yes	373 (62.3)	1.48 (0.79; 2.78)	1.49 (0.75; 2.97)	1.30 (0.63, 2.68)	1.3 (0.62; 2.72)
*Region type*					
Mostly urban	321 (62.2)	Ref	Ref	Ref	Ref
Moderately urban	65 (54.9)	0.74 (0.44; 1.25)	0.88 (0.51; 1.53)	0.88 (0.50, 1.55)	0.81 (0.45; 1.48)
Mostly rural	39 (52.5)	0.67 (0.38; 1.19)	0.79 (0.42; 1.47)	0.79 (0.39, 1.58)	0.81 (0.37; 1.74)
*BMI category*					
Underweight/normal weight	271 (60.2)	Ref	Ref	Ref	Ref
Pre-obesity	111 (61.8)	1.07 (0.61; 1.87)	1.06 (0.62; 1.84)	1.14 (0.65, 2.02)	1.15 (0.65; 2.04)
Obesity	56 (56.7)	0.87 (0.50; 1.52)	1.02 (0.55; 1.91)	0.86 (0.41,1.78)	0.91 (0.43; 1.89)
*Sleep hours*					
<9.5 h/night	70 (70.4)	Ref	Ref	Ref	Ref
9.5–11 h/night	225 (63.8)	0.74 (0.39; 1.43)	0.79 (0.39; 1.61)	0.86 (0.41, 1.80)	0.79 (0.39; 1.6)
≥11 h/night	65 (39.3)	**0.27 (0.14; 0.53)**	**0.30 (0.15; 0.59)**	**0.30 (0.14, 0.63)**	**0.30 (0.15; 0.58)**
*5/day fruits and vegetables*					
No	346 (58.2)	Ref	Ref	Ref	Ref
Yes	95 (67.6)	1.50 (0.94; 2.40)	1.13 (0.68; 1.88)	1.25 (0.65, 2.41)	1.30 (0.66; 2.55)
*Energy (quartiles of intake)*					
1 (lower intake)	96 (55.7)	Ref	Ref	Ref	Ref
2	108 (61.8)	1.34 (0.72, 2.48)	1.50 (0.78, 2.88)	1.13 (0.56, 2.27)	1.13 (0.56, 2.26)
3	123 (66.6)	1.58 (0.83, 3.02)	1.76 (0.91, 3.40)	**2.20 (1.09, 4.44)**	**2.20 (1.09, 4.45)**
4 (higher intake)	114 (57.4)	1.08 (0.59, 1.98)	1.16 (0.65, 2.08)	1.24 (0.64, 2.38)	1.27 (0.64, 2.49)

Boldface indicates statistical significance. BMI, body mass index; SPA, structured physical activity.

∗ Crude model.

† Model adjusted to gender, age, and parents’ education.

‡ Model adjusted to gender, age, parents’ education, sleep hours, and energy intake.

§ Model adjusted to gender, age, parents’ education, sleep hours, and BMI categories.

Table 5 shows that children from parents with tertiary education were more likely to participate in SPA (OR: 6.04; 95% CI: 3.15–11.58). In addition, sleeping at least 11 h/day was associated with lower odds of SPA in about 70% (OR: 0.27; 95% CI: 0.14–0.53) compared with peers sleeping <9.5 h. Results also show that boys have higher odds of participating in SPA than girls (OR, 1.71; 95%CI: 1.02–2.88). Moreover, the association between energy intake and SPA is not linear. Only those classified in the third quartile of intake were significantly more active compared to those in the first quartile (OR, 2.20; 95%CI: 1.09–4.45).

## Discussion

This study describes the prevalence of PA, AOP, and SB among a nationally representative sample of Portuguese children (6–14 years). Data provide updated estimates and novel information to enlighten research and public policies promoting healthy behaviors.

Our findings show that regardless of gender or age group, almost half of the youth failed to meet PA recommendations. Second, there is a substantial decrease in active behaviors but also an increase in SB with age, which may represent 2 cumulated risk factors for illness. Third, on weekends where youngsters are commonly engaged in less PA,^18^ a huge percentage (>70%) of participants exceeded recommended TV time although the guidelines limiting sitting for extended periods.^6^

Overall, the prevalence of spending ≥60 minutes in MVPA/day in 6–14 years old was 54.4%, representing an average of 84 minutes in MVPA. Moreover, as well-established in the literature,^19^ accomplishing PA guidelines significantly decreases with age. Meeting PA guidelines were also associated with energy intake, which may be due to the higher energy requirements of these activity intensities.

Comparing to 2012 data, where 36% of 10–11 years and only 17% of 12–13 years old attained PA guidelines,^10^ our results suggest an increase in overall PA levels among Portuguese youth. This is consistent with HSBC 2014 Report showing that youth engaging in PA for ≥3 times/week increased from 46.7% in 2006 to 51% in 2014.^8^ Still, almost half of youngsters are failing to reach recommended MVPA levels and may be missing its health benefits. The 2013–2014 HBSC survey^9^ showed that at age 11 only 16% of girls and 26% of the boys, in Portugal, reported 60 min/day in MVPA while at 13 the percentage declined for girls (6%) and staged for boys (25%), corroborating our age decline. Similarly, the WHO Global Health Observatory reported 86.7% of 11–17 years old in Portugal did not attain sufficient PA.^20^ Differences found between studies may be due to the diverse age groups considered in each research as PA levels decrease with age;^19^ it may also be due to the diversity of measurement methods employed and outcomes studied that partially explain these inconsistencies. MVPA is likely to be more sporadic and less memorable and quantifiable, especially in children, and therefore underestimated when self-reported compared to objective measures. Moreover, as data in previous studies are not nationwide representative environmental factors such as weather or infrastructures may influence the results.

The importance of AOP is well stated by Active Healthy Kids Global Alliance and WHO;^20^ and its surveillance is crucial for guiding future strategies and interventions because it can reflect the time spent with electronic devices indoors.^21^ Time spent outdoor is a consistent predictor of PA in youth.^22^ When children engage in AOP, they are likely to get more MVPA than during organized activities.^3^ Despite few studies were conducted on this topic there seems to be a decline in outdoor activities over time.^23^ Nonetheless, consistent data on this behavior is still missing worldwide.

Our study is the first to report the national prevalence of AOP. A large percentage of children engaged in ≥60 min/day AOP on weekdays (72%) and on weekends (78%) and this may help explain the results found for MVPA, as these variables are commonly associated.^24^ Likely as MVPA, the prevalence of spending ≥60 min/day in AOP markedly declines with age. Reports from Australia show almost 70% of primary school children (7–12 years) and 50% of secondary school students (12–16) participate in AOP including any non-organized activity of moderate (eg, bike riding and skateboarding) or vigorous-intensity (eg, running, playing tag, or chase).^25^ Belgium's Report Card on PA accounts for 80% of 6–9 years old and 26% to 29% at 10–17 years old engaging in AOP.^26^ Other findings support our data showing more time spent outdoors on weekends than weekdays^27^ despite a systematic review found opposing results.^18^ Our findings may result from children having more freedom of choice on weekends than during the structured school day. Therefore, actions should be taken during school days and hours to allow children to spend recess time outside, increasing AOP and unstructured PA, and decrease sitting time. One of the few Portuguese studies on AOP showed that socioeconomic position can influence children's time spent in outdoor activities, especially on the weekends.^28^ However, our data found no significant differences for parental education, as a proxy for socioeconomic status (SES).

SPA was reported by about 60% of the children. Participation in SPA and average MVPA time increased with higher parental education. Besides time consuming, SPA may imply a fee payment and other costs related to equipment and transportation that may prevent participation from lower SES.^29^ Given the decline in AOP with age and the increase in SB, SPA may be the only opportunity to accumulate activity.^23^ When searching for predictors of SPA targeting children's interests and specificities our results showed that being a boy from parents with secondary or tertiary education increased the odds of participating in SPA while sleeping ≥11 h/day decreases these chances when compared to sleep <9.5 h/day. This is worth exploring in future research. However, one can speculate that SPA is time-consuming and that training hours, typically on weekdays evening and weekend mornings, restrict the time available to sleep. Other findings also showed that more active children have shorter total sleep time than less active children.^30^

Excluding school hours and sleep time, Portuguese youth spend on average 6 h 50 min/day being sedentary, and become more sedentary as they get older (7 h 25 min), which is in line with worldwide trends demonstrating an increase in SB during the transition from primary to secondary school.^31^ This is of relevance given the strong evidence of a relationship between SB and increased risk for obesity and the moderate evidence that links SB to total cholesterol, lower self-esteem, social problems, and low physical fitness in youth.^32^ In line with our results, a survey from 5 European countries estimates children are spending approximately 8 h/day in SB^33^ and objective data from a national study (including school hours) found that 10–11 years old spend more than 8 h on SB and more than 9 h at 14–15 years.^10^ This is alarming as it is one of the highest values found worldwide in this population.

TV viewing was used as a proxy measure for SB as it is the main screen-based behavior and is associated with adverse health outcomes independent of MVPA.^32^ According to our results, excess TV viewing is markedly common, particularly on weekends (71.3%) and would be even more concerning if we had accounted for other electronic devices. Surveillance data from developed countries similarly report a substantial proportion of youngsters to exceed screen time,^31^ making this behavior an important target for interventions. Nonetheless, new insights reveal a possible change in screen time use given the potential of active video games to increase PA.^34^

Gender differences were observed as boys spent more time in SB than girls. Boys also exceeded more frequently 2 h of television on weekdays, which is not surprising as both behaviors may occur concurrently. Consistent with our findings, research shows boys are typically more engaged in TV viewing^35^ and other screen-based activities.^5^ However, studies on gender differences present mixed findings on tracking SB across the lifespan.^31^

### Strengths and limitations

This is the first national representative information on PA and SB on children under 9 years and on AOP providing relevant information for policymakers and health researchers. In addition, the activity diary is a strength compared to questionnaires that may not capture all activity dimensions. Limitations include the study's cross-sectional nature that does not enable to make strong inferences on determinants of the studied behaviors. Misreport-ing energy intake given its self-reported nature.^36^ SPA and TV time were also self-reported, moreover, parents reported younger children's^6–9^ activities but they can be unaware of the activities undertaken during school hours.

## Conclusions

Almost half of the children failed to achieve MVPA recommendations whereas 71% exceeded 2 hours of TV on weekends, suggesting the urgency of intervention measures at a national level. As region of residence, parental education, gender, and age were significantly related either with active and SB, strategies focusing on reducing SB and promoting activity should consider individual, social, and environmental factors.

## Acknowledgments

The authors are grateful to all participants and technical staff who were involved in data collection procedures namely the IAN-AF Consortium: Carla Lopes, Andreia Oliveira, Milton Severo – Faculty of Medicine, University of Porto; Duarte Torres, Sara Rodrigues – Faculty of Nutrition and Food Sciences, University of Porto; Elisabete Ramos, Sofia Vilela – EPIUnit, Institute of Public Health, University of Porto; Sofia Guiomar, Luísa Oliveira – National Health Institute Doutor Ricardo Jorge; Violeta Alarcão, Paulo Nicola – Institute of Preventive Medicine and Public Health, Faculty of Medicine, University of Lisbon; Jorge Mota – CIAFEL, Faculty of Sports, University of Porto; Pedro Teixeira – Faculty of Human Kinetics, CIPER, University of Lisbon, Simão Soares – SilicoLife, Lda, Portugal; Lene Frost Andersen, Faculty of Medicine University of Oslo.

The project had institutional support from the General Directorate of Health (DGS), the Regional Health Administration Departments, the Central Administration of the Health System (ACSS), and from the European Food Safety Authority (CFT/EFSA/DCM/2012/01-C03).

## Funding

This study was conducted in the context of the IAN-AF, National Food, Nutrition and Physical Activity Survey funded by the EEAGRANTS programme-initiatives in Public Health (EEA-GRANTS PT06_00088SI3). AP and JM were supported by grants: FCT: UID/DTP/00617/2019 and AP by SFRH/BPD/ 105071/2014.

## Conflicts of interest

The authors declare no conflicts of interest.
